# Supplementary material for: Constitutive Descriptions and Restoration Mechanisms of a Fe-17Cr Alloy during Deformation at Temperatures of 700–1000 °C
Source: Materials (Basel). 2021 Sep 3;14(17):5022. doi: 10.3390/ma14175022 (PMC8434333; doi:10.3390/ma14175022)
Supplement: Supplementary file 1 [file materials-14-05022-s001.zip › materials-1312332-SI.pdf]

materials

MDPI

*Supplementary*

# Constitutive Descriptions and Restoration Mechanisms of a Fe-17Cr Alloy during Deformation at Temperatures of 700–1000 °C

**Fei Gao [1,2,*], Zilong Gao [3], Qiyong Zhu [3] and Zhenyu Liu [3]**

[1] School of Materials Science and Engineering, Northeastern University, Shenyang 110819, China
[2] Key Laboratory of Lightweight Structural Materials, Liaoning Province, Northeastern University, Shenyang 110819, China
[3] State Key Laboratory of Rolling and Automation, Northeastern University, Shenyang 110819, China; 484320592@qq.com (Z.G.); 1937773035@qq.com (Q.Z.); zhenyuLiu@163.com (Z.L.)
* Correspondence: gaof@mail.neu.edu.cn; Tel.: +86-24-83672224

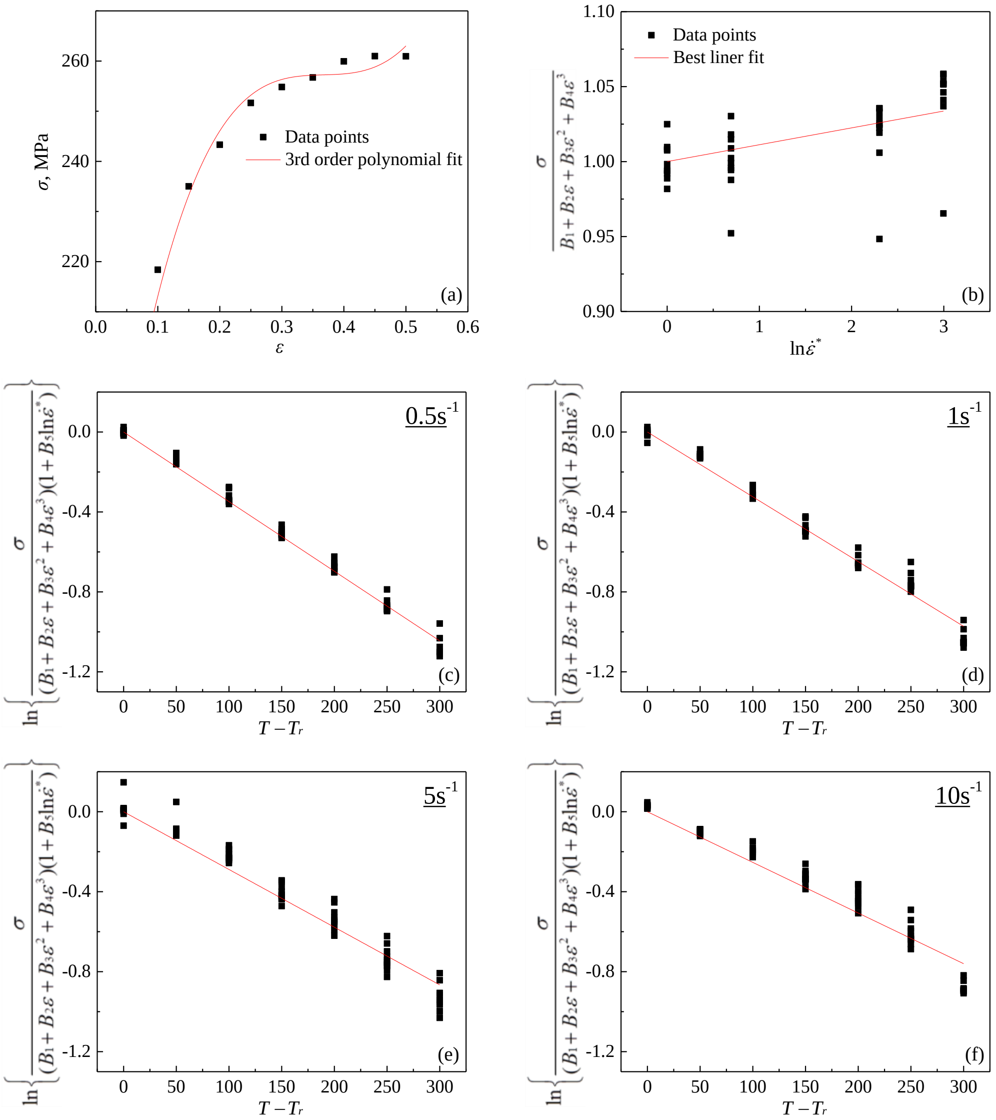

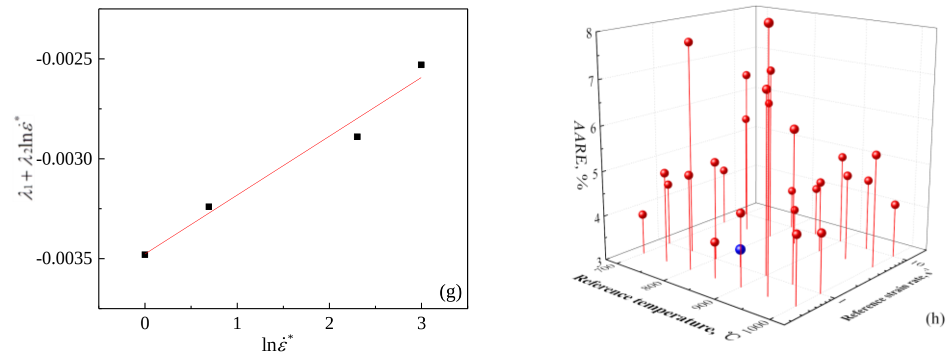


**Figure S1.** Plots used to determine the material parameters at reference temperature of 700 °C and reference strain rate of 0.5 $s^{-1}$ (**a**–**g**) and relationship between *AARE* value and reference condition of the modified Johnson-Cook model for experimental steel (**h**). (**a**) $\sigma - \varepsilon$ plot, (**b**) $\frac{\sigma}{B_1 + B_2\varepsilon + B_3\varepsilon^2 + B_4\varepsilon^3} - \ln\dot{\varepsilon}^*$ plot, (**c**–**f**) $\ln\left\{\frac{\sigma}{\left(B_1 + B_2\varepsilon + B_3\varepsilon^2 + B_4\varepsilon^3\right)\left(1 + B_5\ln\dot{\varepsilon}^*\right)}\right\} - \left(T - T_r\right)$ plot, (**g**) $(\lambda_1 + \lambda_2\ln\dot{\varepsilon}^*) - \ln\dot{\varepsilon}^*$ plot, (**h**) relationship between *AARE* value and reference condition.

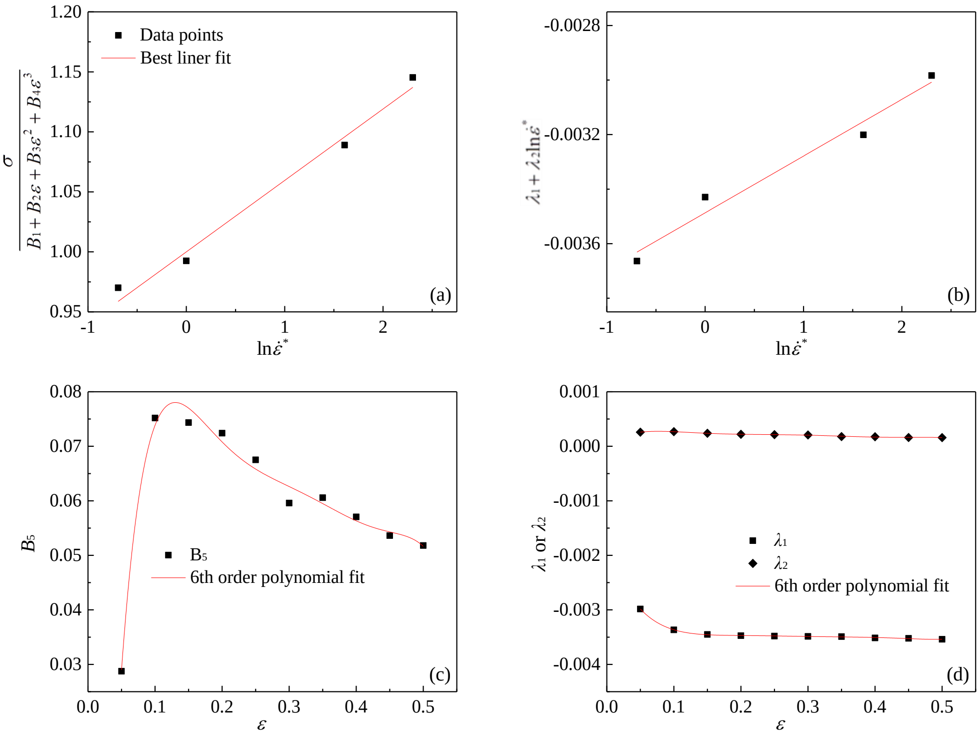


**Figure S2.** Plots used to determine the material parameters $B_5$, $\lambda_1$ and $\lambda_2$ at the strain of 0.3 under the reference temperature of 850 °C and reference strain rate of 1 $s^{-1}$ (a–b) and the strain-dependent material parameters $B_5$, $\lambda_1$ and $\lambda_2$ of modified Johnson-Cook model for the experimental steel (c–d). (**a**) $\frac{\sigma}{B_1 + B_2\varepsilon + B_3\varepsilon^2 + B_4\varepsilon^3} - \ln\dot{\varepsilon}^*$ plot, (**b**) $(\lambda_1 + \lambda_2\ln\dot{\varepsilon}^*) - \ln\dot{\varepsilon}^*$ plot, (**c**) relationship between $B_5$ and $\varepsilon$, (**d**) relationship between $\lambda_1$, $\lambda_2$ and $\varepsilon$.

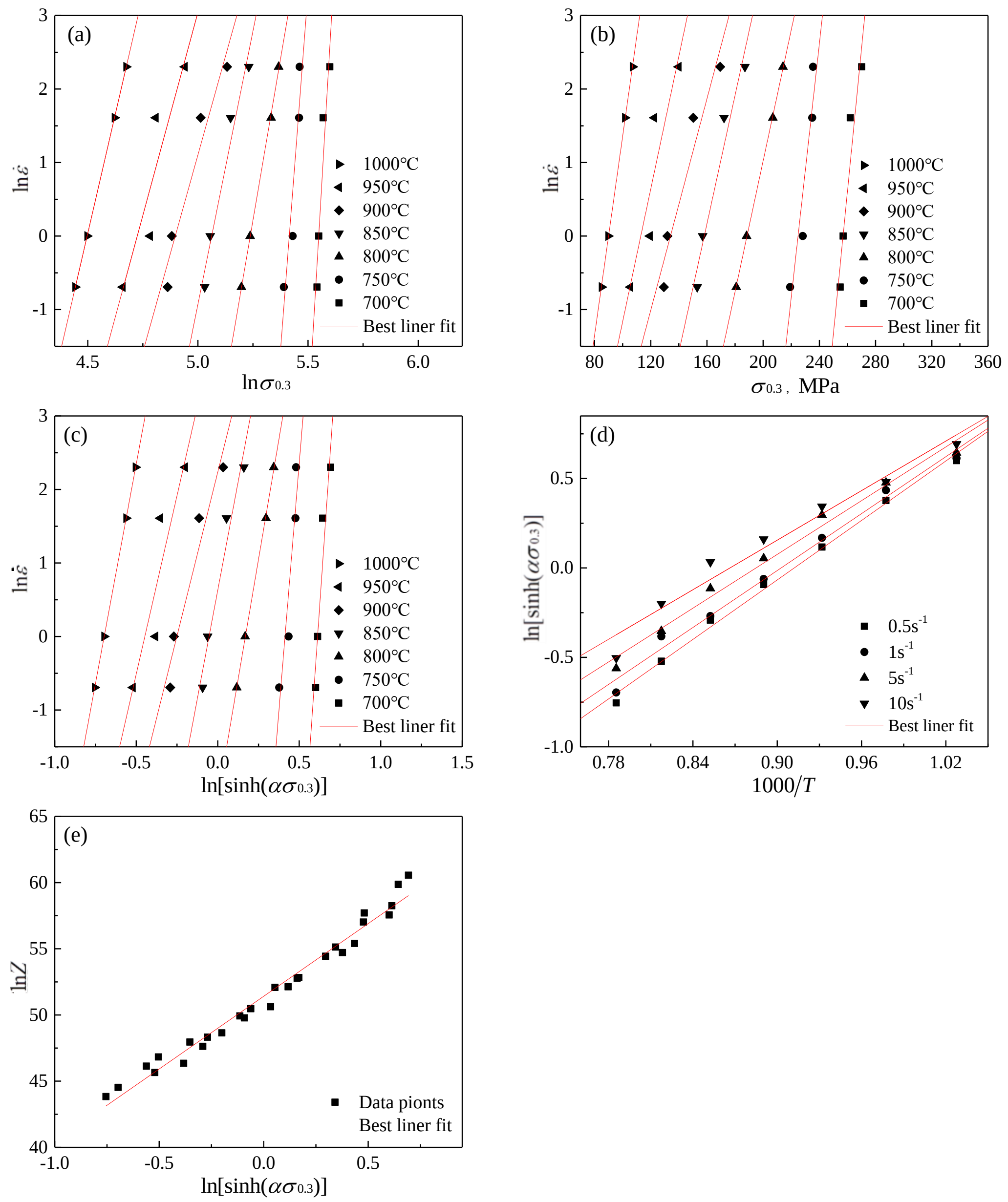


**Figure S3.** Plots used to determine the material parameters *α*, *Q*, *n* and *A* at the strain of 0.3 for modified Arrhenius type model by conventional approach. (**a**) $\ln\dot{\varepsilon}-\ln\sigma_{0.3}$ plot; (**b**) $\ln\dot{\varepsilon}-\sigma_{0.3}$ plot; (**c**) $\ln\dot{\varepsilon}-\ln[\sinh(\alpha\sigma_{0.3})]$ plot; (**d**) $\ln[\sinh(\alpha\sigma_{0.3})]-\frac{1000}{T}$ plot; (**e**) $\ln Z-\ln[\sinh(\alpha\sigma_{0.3})]$ plot.

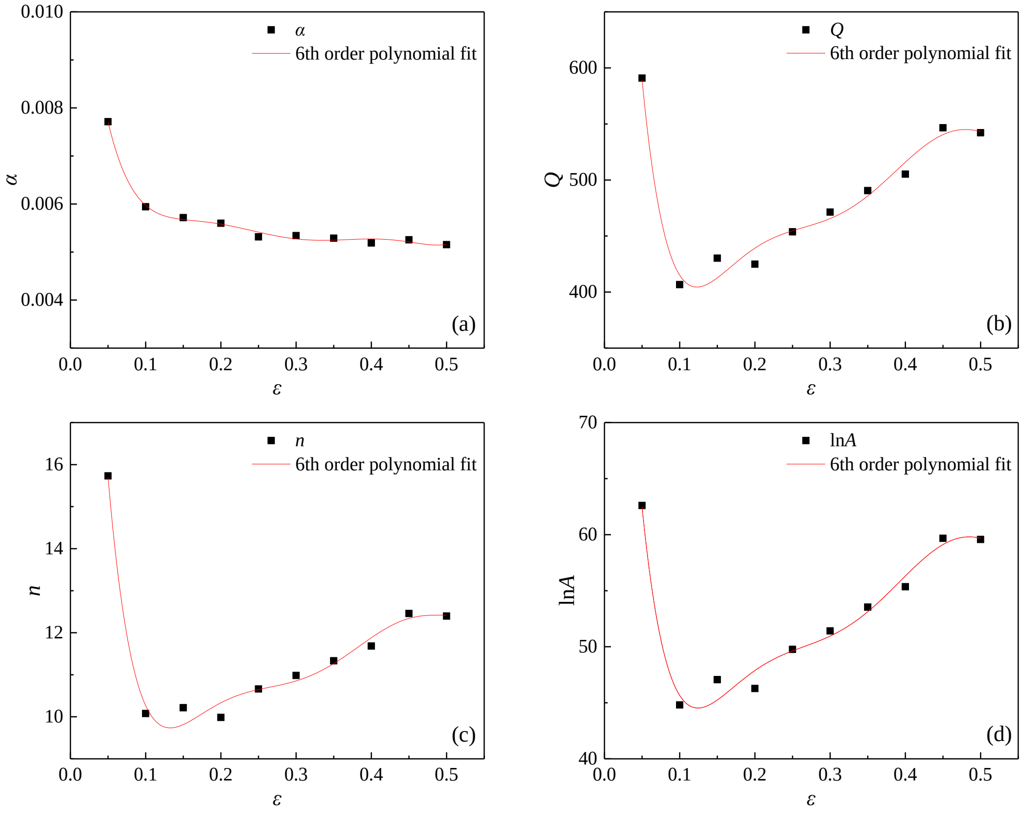


**Figure S4.** Plots used to determine the strain-dependent material parameters $\alpha$, $Q$, $n$ and $A$ for modified Arrhenius type model by conventional approach. (**a**) relationship between $n$ and $\varepsilon$; (**b**) relationship between $n$ and $\varepsilon$; (**c**) relationship between $Q$ and $\varepsilon$; (**d**) relationship between ln$A$ and $\varepsilon$.

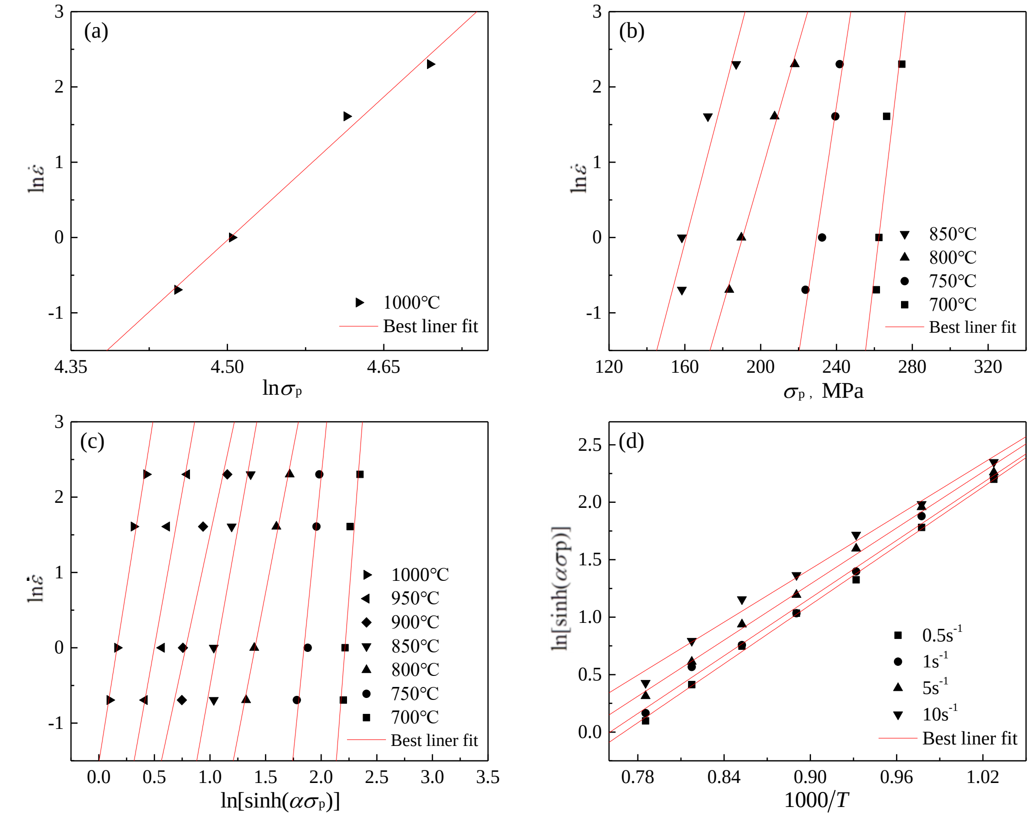

**Figure S5.** Plots used to determine the material parameters $\alpha$ and $Q$ using peak stress for modified Arrhenius type model by proposed approach. (**a**) $\ln\dot{\varepsilon}-\ln\sigma_P$ plot at low stress level; (**b**) $\ln\dot{\varepsilon}-\sigma_P$ plot at high stress level; (**c**) $\ln\dot{\varepsilon}-\ln[\sinh(\alpha\sigma_P)]$ plot; (**d**) $\ln[\sinh(\alpha\sigma_P)]-\frac{1000}{T}$ plot.

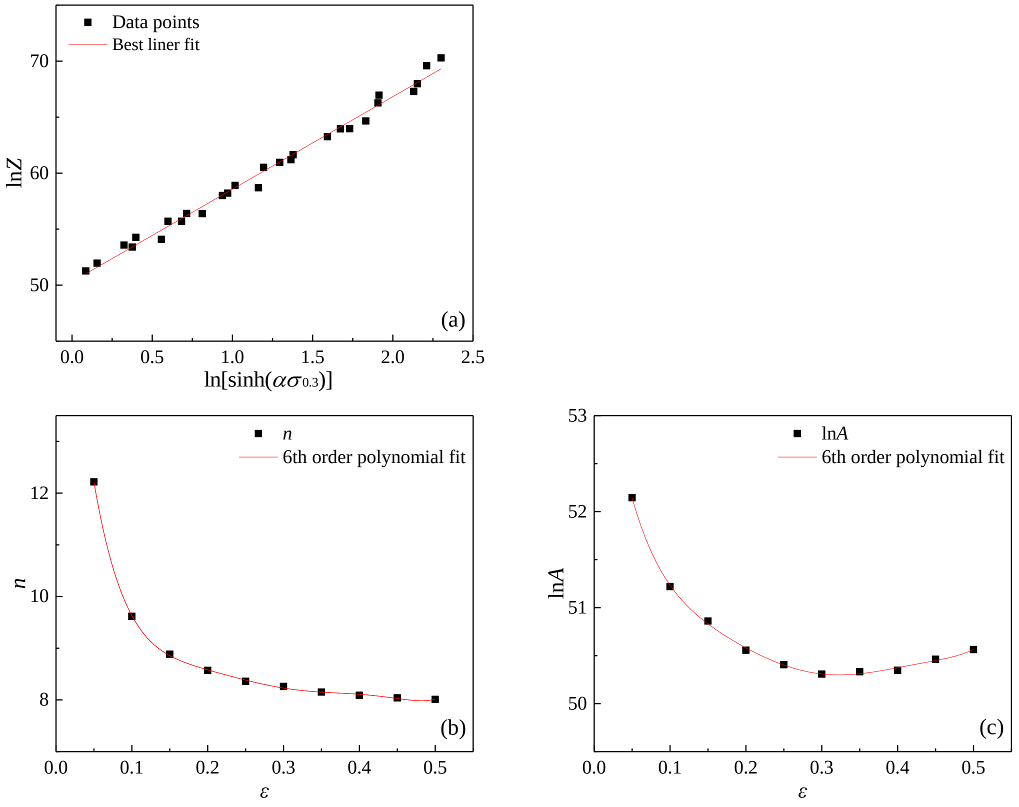


**Figure S6.** Plots used to determine the strain-dependent material parameters $n$ and $A$ for modified Arrhenius type model by proposed approach. (**a**) $\ln Z-\ln[\sinh(\alpha\sigma)]$ plot at the strain of 0.3; (**b**) relationship between $n$ and $\varepsilon$; (**c**) relationship between $\ln A$ and $\varepsilon$.

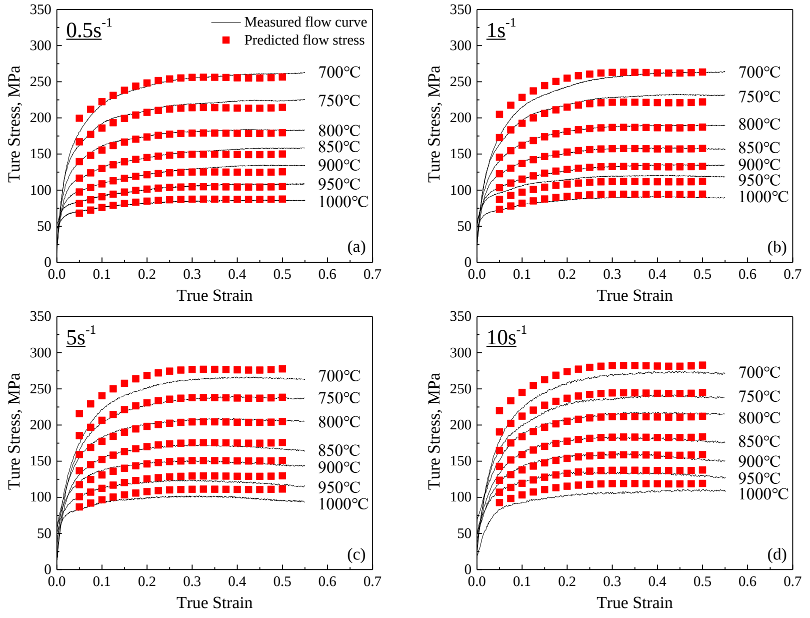

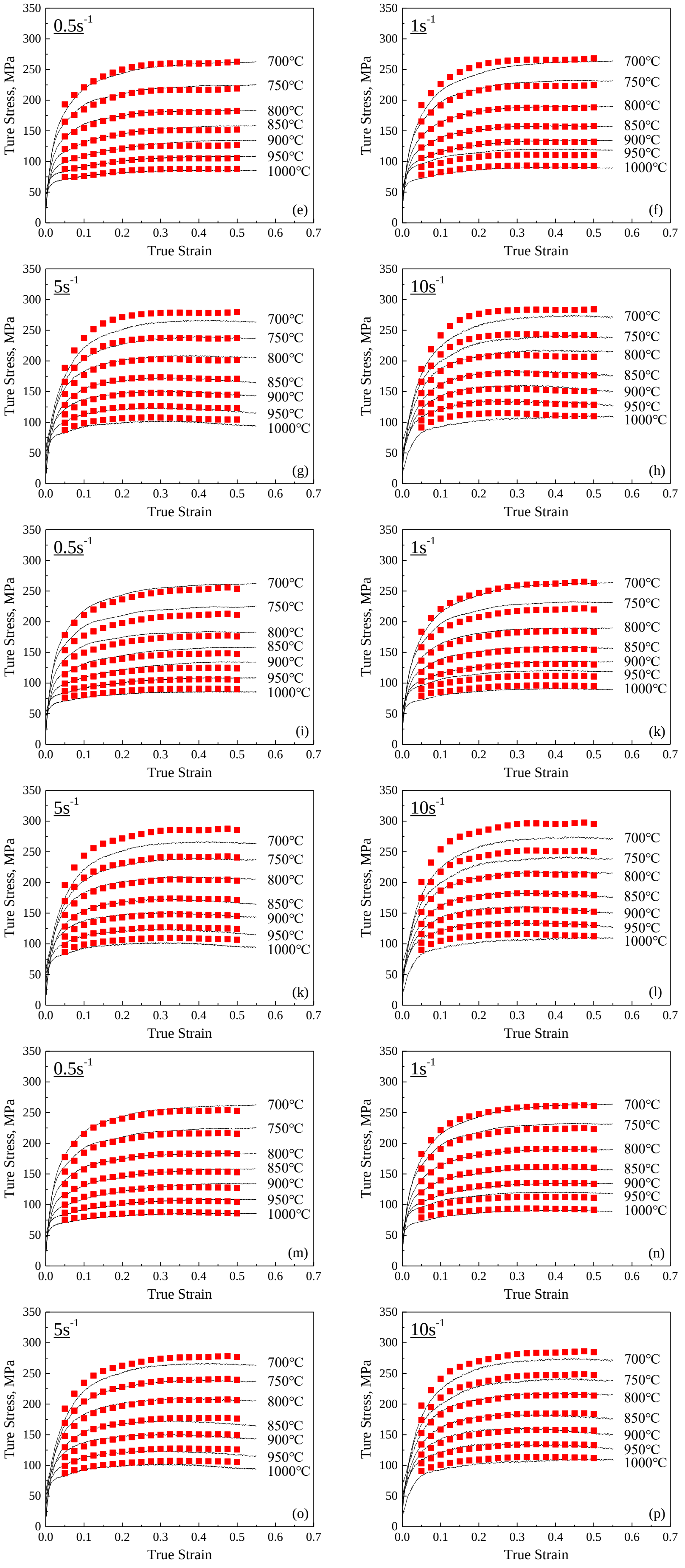
0.5s-1
1s-1
5s-1
10s-1
0.5s-1
1s-1
5s-1
10s-1
0.5s-1
1s-1
5s-1
10s-1
700°C
750°C
800°C
850°C
900°C
950°C
1000°C
(e)
(f)
(g)
(h)
(i)
(k)
(k)
(l)
(m)
(n)
(o)
(p)
Ture Stress, MPa
True Strain
0
50
100
150
200
250
300
350
0.0
0.1
0.2
0.3
0.4
0.5
0.6
0.7

**Figure S7.** Comparison between the measured flow stress curves and predicted flow stress values obtained from the modified Johnson-Cook by conventional approach (a–d), modified Johnson-Cook by considering strain dependency of the parameters (e–h), modified Arrhenius type model by conventional approach (i–l) and modified Arrhenius type model by proposed approach (m–p).
